# Supplementary figures and images for: Multi-method strategies for provenance determination of coarse-grained igneous rocks: Non-destructive, portable, and quantitative approaches
Source: PLoS One. 2025 Jun 3;20(6):e0324058. doi: 10.1371/journal.pone.0324058 (PMC12132978; doi:10.1371/journal.pone.0324058)

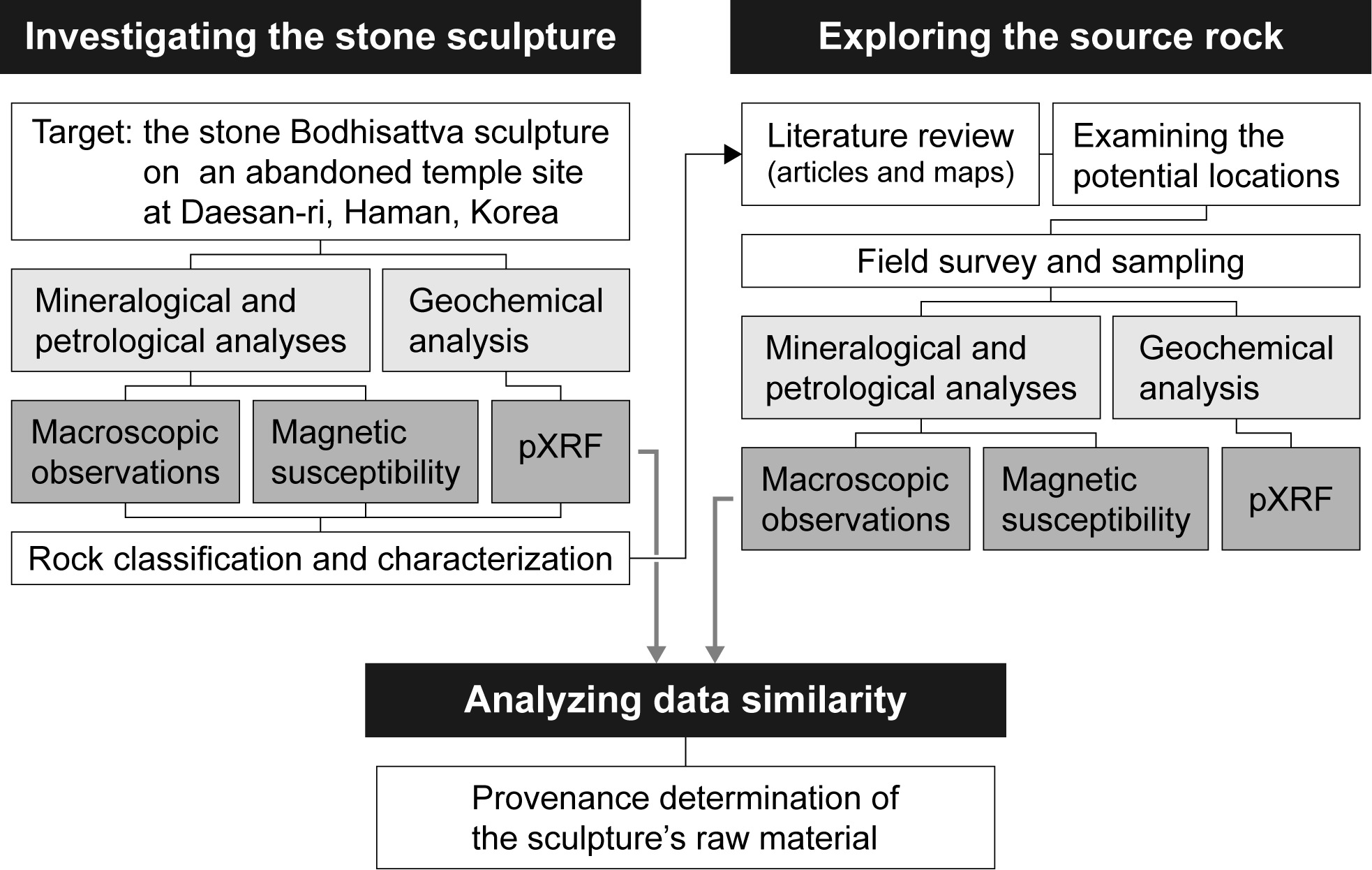

Supplement: S1 Fig — (TIF) [file pone.0324058.s001.tif]

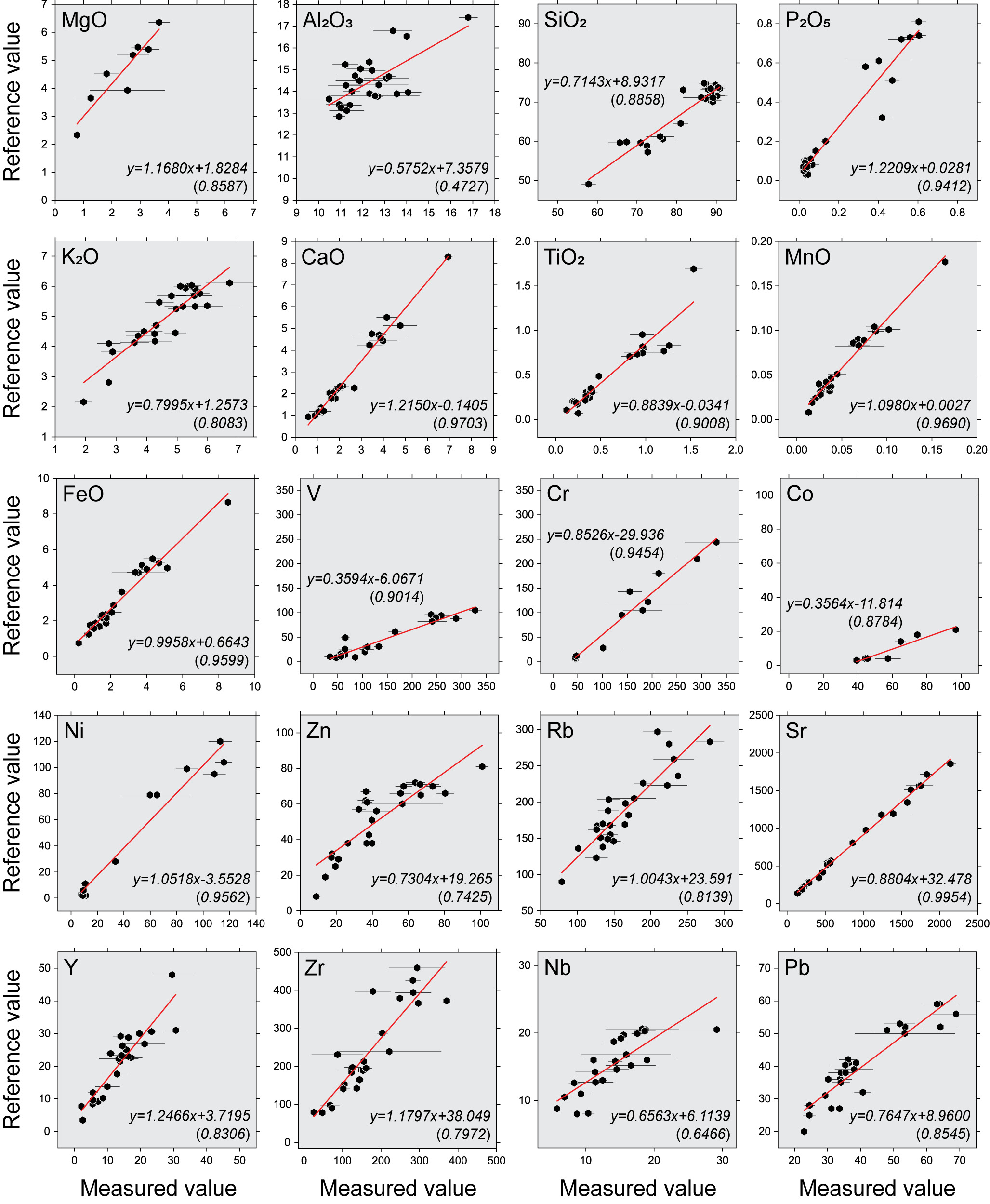

Supplement: S2 Fig — The plots represent the correlation between factory-calibrated pXRF data obtained from in-house reference plutonic rock samples (x-axis) and ICP-MS analysis results for the identical samples (y-axis). Red lines indicate linear regression fits. Horizontal error bars represent the standard deviation of five independent measurements. Displayed equations pertain to calibration models for specific elements, with the correlation coefficient (R2) enclosed in parentheses. Units for chemical concentrations are denoted as weight percent (wt.%) for oxides and parts per million (ppm) for metals. (TIF) [file pone.0324058.s002.tif]

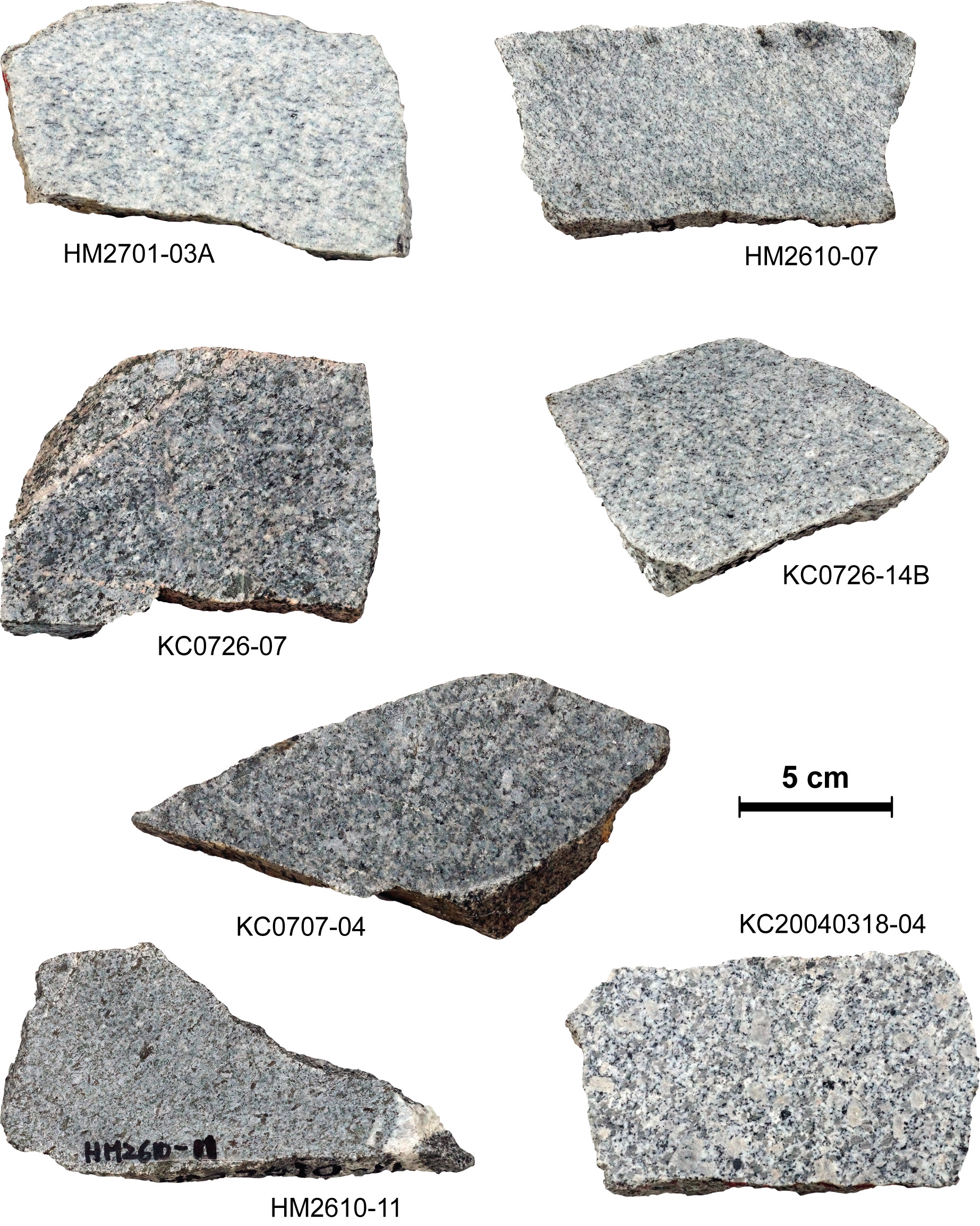

Supplement: S3 Fig — (TIF) [file pone.0324058.s003.tif]
