# Supplementary material for: Multi-method strategies for provenance determination of coarse-grained igneous rocks: Non-destructive, portable, and quantitative approaches
Source: PLoS One. 2025 Jun 3;20(6):e0324058. doi: 10.1371/journal.pone.0324058 (PMC12132978; doi:10.1371/journal.pone.0324058)
Supplement: Attachment — Submitted filename: Response to Reviewers.docx [file pone.0324058.s010.docx]

**Author’s reply for reviewers’ comments on**

**“Multi-method strategies for provenance determination of coarse-grained igneous rocks: Non-destructive, portable, and quantitative approaches”**

(Article no. PONE-D-25-03353-R1 | EMID: 95155b7720cc916e)

We appreciate the valuable comments of two reviewers along with the editor, who spent precious time in dealing with this manuscript. Those are very helpful for improving the manuscript. The authors have carefully considered the comments and tried our best to address every one of them. Our manuscript has been carefully revised with comments of the two reviewers and is much stronger now. In the revised manuscript, all revised changes are highlighted in yellow. In this reply, the reviewers’ comments, authors’ response to the reviewers, and changes in the revised manuscript are colored as black, blue, and red, respectively. Please note that all page and line numbers refer to the revised manuscript.

Additionally, we wish to inform that this document includes responses to additional edits requested to clarify licensing issues concerning the sources of some figures included in the manuscript.

**Journal Requirements (updated):**

1. Thank you for providing information about the source of the map images in Figure 1 (https://data.kigam.re.k). However, please note that any shapefiles, map data, basemaps, etc. copyrighted under a CC BY-NC license cannot be published in PLOS ONE under the CC BY 4.0 license without permission from the copyright owner(s). Unfortunately, the CC BY NC license is more restrictive than the CC BY 4.0 license that PLOS ONE utilizes.

***Authors’ reply*:** The authors obtained permission from the institution holding the copyright for the source of the map images in Figure 1. We have uploaded the completed permission form, titled “content-permission-form-PONE-D-25-03353.pdf”, along with the revised manuscript. Please confirm. In addition, the following text has been added to the figure caption of Figure 1:

• [Pg3, Ln58–61 in revised manuscript]

… **areas in South Korea.** The map is republished from several digital geological maps [31–41] under a CC BY license, with permission from the Geoscience Data Center of the Korea Institute of Geoscience and Mineral Resources (https://data.kigam.re.kr), original copyrights 1963, 1964, 1969, 1972, 1975, 1983, and 2021.

2. Please confirm if the map image in Figure 6 is from the same source as the map images in Figure 1. If not, please confirm the source of the the map image (basemap, shapefile, map data, etc.) in Figure 6.

***Authors’ reply*:** The authors confirm that the source of the map image used in Figure 6A is the same as that in Figure 1. Therefore, a CC BY license can be applied without any problem. As in Figure 1, text related to the license has been added to the figure caption of Figure 6.

• [Pg13, Ln297–299 in revised manuscript]

… the mean of the relevant variables. The map is republished from several digital geological maps [31–41] under a CC BY license, with permission from the Geoscience Data Center of the Korea Institute of Geoscience and Mineral Resources (https://data.kigam.re.kr), original copyrights 1963, 1964, 1969, 1972, 1975, 1983, and 2021.

3. Please explain where the authors obtained the images in Figures 2 and S3 in your submission. If the authors created these images themselves, please confirm this in your response.

***Authors’ reply*:** The authors confirm that Figures 2 and S3 are based on photographs taken by the first author, Bongsu Chang, and have not been previously published or copyrighted.

**Journal Requirements:**

1. Please ensure that your manuscript meets PLOS ONE's style requirements, including those for file naming. The PLOS ONE style templates can be found at [URLs].

***Authors’ reply*:** Done. The authors have confirmed that the submitted manuscript complies with PLOS ONE’s style guidelines, as outlined in the linked templates, and have made several minor modifications detailed below.

• [Pg1, Ln8 in revised manuscript]

* Corresponding author …

• [Pg4, Ln63 in revised manuscript]

Materials and methods …

• [Supporting information]

The names of the supporting information files were updated to correspond with the captions in the manuscript (e.g., S1-S3_figs.docx and S1-S5_tables.xlsx).

2. In your manuscript, please provide additional information regarding the specimens used in your study. Ensure that you have reported human remain specimen numbers and complete repository information, including museum name and geographic location.

***Authors’ reply*:** Done. The authors revised the manuscript and supplementary tables to provide geographic location information (GPS coordinates) for the stone sculpture and thirteen outcrops. In addition, a statement regarding the permits for this study has been added to the manuscript.

S1 Table. Geographic location information (GPS coordinates) of thirteen outcrops.

| **Site** | **GPS coordinates** | |
| --- | --- | --- |
|  | **Latitude** | **Longitude** |
| HA01 | 35° 23' 09.2898" N | 128° 36' 51.1086" E |
| HA02 | 35° 21' 27.1800" N | 128° 36' 03.5460" E |
| HA03 | 35° 20' 12.5874" N | 128° 34' 19.3038" E |
| HA04 | 35° 18' 16.8474" N | 128° 34' 23.5950" E |
| HA05 | 35° 11' 33.9612" N | 128° 31' 37.6710" E |
| HA06 | 35° 11' 00.5280" N | 128° 32' 19.1508" E |
| HA07 | 35° 11' 49.4514" N | 128° 26' 06.7770" E |
| HA08 | 35° 06' 23.7198" N | 128° 20' 04.5492" E |
| HA09 | 35° 06' 59.0682" N | 128° 21' 45.2370" E |
| HA10 | 35° 11' 46.7730" N | 128° 23' 13.7394" E |
| HA11 | 35° 11' 57.2244" N | 128° 22' 45.6708" E |
| HA12 | 35° 22' 53.4462" N | 128° 12' 59.1048" E |
| HA13 | 35° 14' 41.4996" N | 128° 17' 25.0260" E |

• [Pg4, Ln73 in revised manuscript]

… (35° 15' 18.6430" N, 128° 25' 43.3892" E), located in the southeastern Korean Peninsula …

• [Pg4, Ln80–82 in revised manuscript]

… for restoration. All necessary permits were obtained for the described study from the Cultural Heritage Officer at the Haman County Office, and the fieldwork was conducted in compliance with all relevant regulations.

• [Pg5, Ln100 in revised manuscript]

… 25 km of the sculpture (Fig 1 and S1 Table). A subsequent field trip …

• [Pg17, Ln470 in revised manuscript]

S1 Table. Geographic location information (GPS coordinates) of thirteen outcrops.

• Note: the numbering of the supplementary tables has been adjusted following the addition of S1 Table, and the corresponding sections of the manuscript and supplementary files have been updated.

3. Thank you for stating the following financial disclosure: This work was supported by the National Research Foundation of Korea (NRF) grant funded by the Korea government (MSIT) (grant no. RS-2024-00345589, recipient YJL). Please state what role the funders took in the study. If the funders had no role, please state: "The funders had no role in study design, data collection and analysis, decision to publish, or preparation of the manuscript." If this statement is not correct you must amend it as needed. Please include this amended Role of Funder statement in your cover letter; we will change the online submission form on your behalf.

***Authors’ reply*:** The funders had no role in study design, data collection and analysis, decision to publish, or preparation of the manuscript. Since the aforementioned statement requires no amendments, it is not included in the cover letter.

4. Please include your full ethics statement in the ‘Methods’ section of your manuscript file. In your statement, please include the full name of the IRB or ethics committee who approved or waived your study, as well as whether or not you obtained informed written or verbal consent. If consent was waived for your study, please include this information in your statement as well.

***Authors’ reply*:** According to PLOS ONE's submission guidelines, ethics statements are required in the Methods section for studies involving human subjects. Since this study does not involve human subjects, no such statement was provided.

5. We note that Figure 1 in your submission contain [map/satellite] images which may be copyrighted. All PLOS content is published under the Creative Commons Attribution License (CC BY 4.0), which means that the manuscript, images, and Supporting Information files will be freely available online, and any third party is permitted to access, download, copy, distribute, and use these materials in any way, even commercially, with proper attribution. For these reasons, we cannot publish previously copyrighted maps or satellite images created using proprietary data, such as Google software (Google Maps, Street View, and Earth). For more information, see our copyright guidelines: http://journals.plos.org/plosone/s/licenses-and-copyright.

***Authors’ reply*:** Figure 1 is a derivative work produced by the first author (Bongsu Chang) using several digital geological maps available under a CC BY-NC License from the Geo Big Data Open Platform (<https://data.kigam.re.kr>). Because this license permits the free selection of a license for derivative works, the figure does not conflict with PLOS ONE’s CC BY publication policy. To alleviate any concerns, the figure caption now includes the appropriate citations for the map’s creator and source, and the reference list has been modified accordingly.

• [Pg3, Ln58–61 in revised manuscript]

… **areas in South Korea.** The map is republished from several digital geological maps [31–41] under a CC BY license, with permission from the Geoscience Data Center of the Korea Institute of Geoscience and Mineral Resources (https://data.kigam.re.kr), original copyrights 1963, 1964, 1969, 1972, 1975, 1983, and 2021.

6. Please review your reference list to ensure that it is complete and correct. If you have cited papers that have been retracted, please include the rationale for doing so in the manuscript text, or remove these references and replace them with relevant current references. Any changes to the reference list should be mentioned in the rebuttal letter that accompanies your revised manuscript. If you need to cite a retracted article, indicate the article’s retracted status in the References list and also include a citation and full reference for the retraction notice.

***Authors’ reply*:** The authors reviewed the reference list to ensure its completeness and correctness. No retracted articles were cited, and all changes have been documented in the rebuttal letter accompanying the revised manuscript.

**Reviewer #1:**

**General comments:**

This is a fine study on this one stone sculpture, using an appropriate multi-method approach.

***Authors’ reply*:** Thank you for your positive general comment.

**Specific comments:**

1. You should make clear the sequence of analytical methods used, and if there was any selection in reducing the number of tests.

***Authors’ reply*:** Thank you for your constructive comment. In response to your suggestion, the authors have added a supplementary figure (S1 Fig) showing the sequence of analytical methods in a flowchart. Of course, supplementary figure caption numbers were adjusted to account for the newly added figure.

Regarding comment concerning the number of tests, we clarify that no such selection was made. In designing the study, we considered various petrographic, mineralogical, and geochemical analytical methods for rock characterization and employed all techniques available with our equipment that met the criteria of being non-destructive, portable, and quantitative.

S1 Fig. Flowchart showing the sequence and structure of the analytical methods used in this study.

• [Pg5, Ln96 in revised manuscript]

… structured into three stages (S1 Fig). The first stage involves …

• [Pg17, Ln467 in revised manuscript]

S1 Fig. Flowchart showing the sequence and structure of the analytical methods used in this study.

2. It should be made clear that pXRF calibration is needed for potential comparison of data between labs and different instruments. In your case, the analyses of multiple spots supported the reliability of your results and interpretation, while accuracy of your elemental data is good for your report for future studies.

***Authors’ reply*:** Thank you. The point you raised is fundamental to the need for pXRF calibration. The authors have added a sentence to the revised manuscript to address this issue.

• [Pg6, Ln130–132 in revised manuscript]

… As previously noted, the target rock’s coarse-grained texture … for correcting elemental data [11,13,14]. Moreover, pXRF calibration is essential for comparing data across different laboratories and instruments. To meet these requirements, …

3. Where you discuss Figure 5 (lines 197 onward), need to make clear what the different symbols (open squares, filled circles, filled hexagons) mean. For the pXRF elemental analysis, indicate which elements are represented in the bar graph.

***Authors’ reply*:** First, detailed information on the symbols used in the figure is provided in the existing caption, and a brief sentence has been added to the revised manuscript to note this. Next, a sentence has been included in the caption to clarify that the cumulative counts in the bar graph correspond to the elements presented in Figure 4. Additionally, the results in Figure 4 indicate which elements at each site match the selection criteria.

• [Pg9, Ln205 in revised manuscript]

… and magnetic susceptibility analysis. Detailed information on the symbols and bar graph is provided in the caption.

• [Pg10, Ln213–216 in revised manuscript]

… where magnetic susceptibility is matched. ~~Cumulative number of matches for individual oxide compositions is displayed in a horizontal bar graph.~~ A horizontal bar graph displays the cumulative number of matches for each oxide composition, reflecting the geochemical similarity among the eight elements presented in Figure 4.

4. In Figure 6A, where is the sculpture (asterisk)? It should be as clear as you have for 6B and 6C. Or not in the range of this map?

***Authors’ reply*:** Thank you for your detailed comment. The authors have added a star-shaped symbol in Figure 6A to indicate the location of the sculpture.

Fig 6. Constraining the provenance of source rock by analyzing spatial variations in the chemical composition of plutonic bodies. (A) Spatial distribution of …

5. In Conclusion, indicate the distance from where the sculpture is to its geological source.

***Authors’ reply*:** Thank you for your comment. Identifying the quarry site for the stone sculpture’s source rock was a primary objective of this study. Once achieved, this finding allows for inferences about the scale of labor involved and insights into contemporary social life. Although this extended discussion was not included in this submission, we have added relevant content to the revised manuscript to acknowledge its socio-cultural significance.

• [Pg13–14, Ln311–313 in revised manuscript]

Consequently, the source rock used in the sculpture appears to have been quarried from a site approximately 20 km away (in a straight line) from its current location. Considering the rugged terrain, the actual transportation route is estimated to have spanned about 35 km, following valleys, inland waterways, and the southern sea. Our methodology introduces …

6. Are there any other sculptures at this site or elsewhere in the Haman area that might be tested in the future? This would be very informative for any patterns in the use of particular stone for these and other sculptures. Possible future research?

***Authors’ reply*:** Certainly. In the Haman region, there are additional stone sculptures beyond the subject of this study; however, no investigations have yet been planned for them. Should such research be undertaken in the future, as you noted, an analysis of the use of specific stone in these and other sculptures would likely yield valuable insights. Thank you for your interest and encouragement.

7. Minor corrections:

***Authors’ reply*:** All of the minor corrections you noted have been incorporated into the revised manuscript.

• [Pg2, Ln23 in revised manuscript]

… Plutonic rocks have served as common resources for ~~the~~ artifact creation since prehistoric times. They were highly valued …

• [Pg2, Ln25 in revised manuscript]

… the gradual cooling of magma in a subsurface, results in a coarse-crystalline …

• [Pg4, Ln72 in revised manuscript]

The target of this study, the stone Bodhisattva sculpture …

• [Pg5, Ln99 in revised manuscript]

… a literature review including geological maps enabled us to identify …

• [Pg8, Ln171–172 in revised manuscript]

… approximately 5% on average, with and absolute error … the relative error averaged about 13%.

• [Pg9, Ln197 in revised manuscript]

… multi-analytical investigations in the provenance study. In …

• [Pg9, Ln207 in revised manuscript]

… Following the HA09 site, which matched in eight criteria, …

• [Pg10, Ln224–235 in revised manuscript]

… could be narrowed down in each case (Table 1). ~~When each method was applied individually (diagonal part of the table), macroscopic observations narrowed the possibilities to three out of thirteen cases, magnetic susceptibility to four, and pXRF alone identified a single outcrop. Combining the other two methods, excluding pXRF, still did not isolate a single candidate. Therefore, this study demonstrated that successful source identification can be achieved solely through the analysis of geochemical properties using pXRF.~~ Table 1 is presented as a grid showing how each of the three analytical methods (i.e., macroscopic observations, pMS, and pXRF) may be applied individually or in combination. Each cell indicates the number of outcrops selected by that particular combination of methods. The diagonal cells show the number of outcrops meeting the selection criteria when each method is applied alone, whereas the off-diagonal cells show the number selected when two methods are combined. Specifically, macroscopic observations narrowed the selection to three of thirteen outcrops, magnetic susceptibility to four, and pXRF alone to one. In contrast, combining the other two methods (excluding pXRF) did not isolate a single outcrop. Consequently, this study demonstrates that geochemical analysis through pXRF alone can successfully identify a source. These findings emphasize …

• [Pg11, Ln242–245 in revised manuscript]

… **the number of outcrops selected for each combination.** The diagonal cells show how many outcrops were passed selection criteria by that single method, while the off-diagonal cells show how many outcrops were selected by the combination of the two analytical methods where the row and column intersect.

• [Pg11, Ln250 in revised manuscript]

… However, when the volume of a selected outcrop is too small or fractured to use …

• [Pg13, Ln302 in revised manuscript]

… the provenance of coarse-grained igneous rock used in this sculpture by analyzing …

**Reviewer #2:**

**General comments:**

The research work seems novel to me, as it helps to carry out non-destructive research studies on archaeological materials composed of plutonic rocks with visible crystal sizes. The authors have been able to tackle the problem, using several different methods and understanding the genesis of these plutonic rocks. The text is very well articulated and in very clear language. Below I point out some formal issues regarding the text:

***Authors’ reply*:** Thank you for your kind general comments. As you are aware, if sufficient destructible samples were available and analytical methods were unrestricted, even more accurate data could be obtained. Nonetheless, our study represents an effort to establish a foundation for unequivocal, objective conclusions by providing the most quantitative analysis possible, even when limited to non-destructive methods for preservation purposes. We are delighted to find that you share this perspective.

Fig 1. Geologic map of the study area including Haman and surrounding areas in South Korea. The map is republished from several digital geological maps [31–41] under a CC BY license, with permission from the Geoscience Data Center of the Korea Institute of Geoscience and Mineral Resources …

**Specific comments:**

1. Figure 1 should have a world map to later locate the map of Korea. To avoid confusion, the blue color of the map should be written: Korean Sea.

***Authors’ reply*:** Thank you for your valuable comment to improve the manuscript. In response, the authors have added a world map to Figure 1 and labeled the blue area as “South Sea”. Please refer to Figure 1 on the previous page for the final result.

2. In Figure 6A, the location of the star that indicates the sculpture is not clearly visible.

***Authors’ reply*:** Thank you for your detailed comment. The authors have added a star-shaped symbol in Figure 6A to indicate the location of the sculpture.

Fig 6. Constraining the provenance of source rock by analyzing spatial variations in the chemical composition of plutonic bodies. (A) Spatial distribution of …

3. A figure with more detailed photographs of the texture and size of crystals is missing (as far as possible), comparing the rock of the sculpture with the source rock.

***Authors’ reply*:** The authors appreciate your perspective; however, please understand that it is not possible to provide more detailed photographs than those in Figure 2 under current constraints. Nevertheless, the provided photographs meet our macroscopic observational criteria for verifying the presence or absence of mafic enclaves and pinkish alkali feldspars.

4. Has the quarry or signs of quarrying been found? I imagine that this point in question is unlikely. Unless this rock was chosen to build other buildings.

***Authors’ reply*:** No literature on quarrying was found, nor were any traces of quarrying detected during the field survey. Unless the quarry was exceptionally large, any remnants would likely have disappeared. Furthermore, government reforestation efforts following the Korea War (1950–1953) have largely covered the mountainous areas with vegetation, further obscuring any historical traces.

5. Are the sedimentary layers widely distributed around the sculpture formed in terrestrial environments during the Cretaceous period? Are they not typical of marine environments?

***Authors’ reply*:** The sedimentary layers in question are part of the Gyeongsang Basin, the largest non-marine Cretaceous sedimentary basin in Korea and Japan. It formed through sinistral strike-slip movement induced by the oblique subduction of the paleo-Pacific plate beneath the eastern Asian continental margin and has evolved over approximately sixty million years [1-3].

6. Is the plutonic rock a tonalite or granodiorite? The mineralogy suggests that it is an intermediate facies.

***Authors’ reply*:** Strictly speaking, the modal mineralogical compositions have not been determined, limiting the precise classification of the plutonic rock as either tonalite or granodiorite. However, given the minimal or absent alkali feldspar and the predominance of quartz and plagioclase, the rock is expected to plot in the tonalite (or granodiorite) field on a QAPF diagram. As you noted, it is also plausible that the rock represents an intermediate facies between tonalite and granodiorite.

7. On line 179 it says: “This pattern is considered to be the results of the differentiation process during magma cooling that formed the plutons”. This is true, coinciding with the observation made previously on the presence of enclaves, the abundance of biotite and hornblende, and the scarcity of alkaline feldspar. In principle, more mafic minerals accumulate in the magmatic differentiation process. The existence of biotite and hornblende (rich in Fe and Ti), are a strong argument to explain the high values of FeO and TiO2 existing in the rock corresponding to the sculpture. In parallel, the low presence of alkaline feldspar in this same rock is responsible for the low content of K2O and SiO2. It would have been very good to be able to check the reaction textures in the different enclaves, an issue that is not the subject of this research, since it is only possible through destructive petrographic methods.

***Authors’ reply*:** Although not within the scope of this study, the authors appreciate your intriguing petrological insight. While we currently lack the capacity to undertake a detailed investigation of magma evolution in this region, we will verify the reaction texture you mentioned in future research to ensure they serve as valuable observational evidence.

**References**

1. Lee TH, Park KH, Yi K. Nature and evolution of the Cretaceous basins in the eastern margin of Eurasia: A case study of the Gyeongsang Basin, SE Korea. J Asian Earth Sci. 2018; 166:19–31. doi: 10.1016/j.jseaes.2018.07.004
2. Hwang BH, Son M, Yang K, Yoon J, Ernst WG. Tectonic evolution of the Gyeongsang Basin, southeastern Korea from 140 Ma to the present, based on a strike-slip and block rotation tectonic model. Int Geol Rev. 2008; 50:343–63. doi: 10.2747/0020-6814.50.4.343
3. Lee C, Ryu IC, Shinn YJ. U-Pb ages of detrital zircons in Lower Cretaceous non-marine successions of the Gyeongsang Basin, Northeast Asia: Implications for sediment provenance. Sediment Geol. 2017; 353: 125–38. doi: 10.1016/j.sedgeo.2017.03.008
31. Kang PJ, Park SW. Geological map of Korea (Sam Ga Sheet, 1:50,000). Geological and Mineral Institute of Korea. 1975 Dec 30. doi: 10.22747/data.20211214.4649
32. Choi SO, Yeo SC. Geological map of Korea (Nam Ji Sheet, 1:50,000). Geological Survey of Korea. 1972 Dec 30. doi: 10.22747/data.20211221.4674
33. Kim NJ, Lee HK. Geological map of Korea (Yongsan Sheet, 1:50,000). Geological Survey of Korea. 1964 Dec 31. doi: 10.22747/data.20211203.4616
34. Kim OJ, Yoon S, Gil YJ. Geological map of Korea (Jinju Sheet, 1:50,000). Geological Survey of Korea. 1969 Dec 30. doi: 10.22747/data.20211022.4500
35. Choi YK, Kim TY. Geological map of Korea (Uiryong Sheet, 1:50,000). Geological Survey of Korea. 1963 Dec 30. doi: 10.22747/data.20211022.4499
36. Kim JH, Kim JT. Geological map of Korea (Masan Sheet, 1:50,000). Geological Survey of Korea. 1963 Dec 30. doi: 10.22747/data.20211203.4617
37. Choi, YK. Geological map of Korea (Sacheon Sheet, 1:50,000). Geological Survey of Korea. 1969 Dec 30. doi: 10.22747/data.20211022.4537
38. Park NY, Chi JM. Geological map of Korea (Chindong-ni Sheet, 1:50,000). Geological Survey of Korea. 1963 Dec 30. doi: 10.22747/data.20211104.4575
39. Hwang SK, Ko K, Ahn GM. Geological map of Korea (Jinhae-Dongdumal Sheet, 1:50,000). Korea Institute of Geoscience and Mineral Resources. 2021. doi: 10.22747/data.20231115.5203
40. Chi JM, Kim HS, Oh IS, Kim HC. Geological map of Korea (Samcheonpo Sheet, 1:50,000). Korea Institute of Energy and Resources. 1983. doi: 10.22747/data.20211022.4501
41. Chang TW, Hwang SK, Lee DW, Oh IS, Kim HC, Kim EH. Geological map of Korea (Chung Mu Sheet, 1:50,000). Korea Institute of Energy and Resources. 1983. doi: https://doi.org/10.22747/data.20211221.4676
